# Potential therapeutic target for aging and age-related neurodegenerative diseases: the role of acid sphingomyelinase

**DOI:** 10.1038/s12276-020-0399-8

**Published:** 2020-03-13

**Authors:** Min Hee Park, Hee Kyung Jin, Jae-sung Bae

**Affiliations:** 1 KNU Alzheimer’s disease Research Institute, Kyungpook National University, Daegu, 41566 South Korea; 2 Department of Physiology, Cell and Matrix Research Institute, School of Medicine, Kyungpook National University, Daegu, 41944 South Korea; 3 Department of Biomedical Science, BK21 Plus KNU Biomedical Convergence Program, Kyungpook National University, Daegu, 41944 South Korea; 4 Department of Laboratory Animal Medicine, College of Veterinary Medicine, Kyungpook National University, Daegu, 41566 South Korea

**Keywords:** Diseases of the nervous system, Blood-brain barrier

## Abstract

Aging, which is associated with age-related changes in physiological processes, is the most significant risk factor for the development and progression of neurodegenerative diseases, including Alzheimer’s disease and Parkinson’s disease. Accumulating evidence has indicated that sphingolipids are significant regulators that are associated with pathogenesis in aging and several age-related neurodegenerative diseases. In particular, abnormal levels of acid sphingomyelinase (ASM), one of the significant sphingolipid-metabolizing enzymes, have been found in the blood and some tissues under various neuropathological conditions. Moreover, recent studies have reported the importance of ASM as a critical mediator that contributes to pathologies in aging and age-related neurodegenerative diseases. In this review, we describe the pathophysiological processes that are regulated by ASM, focusing on the age-related neurodegenerative environment. Furthermore, we discuss novel insights into how new therapeutics targeting ASM may potentially lead to effective strategies to combat aging and age-related neurodegenerative diseases.

## Age-related neurodegenerative diseases: role of enzyme crucial to membrane function

Targeting a critical enzyme involved in the metabolism of cellular membrane molecules could help treat age-related neurodegenerative diseases. Aging is a key risk factor for the development of neurodegenerative diseases, including Alzheimer’s and Parkinson’s. Changes to the metabolism of sphingolipids, a group of bioactive cell membrane molecules, are recognised as a potential trigger of neurodegeneration. Abnormal expression levels of an enzyme called acid sphingomyelinase (ASM), which is involved in sphingolipid metabolism, have been found in patients with age-related diseases. Jae-sung Bae and co-workers at Kyungpook National University in Daegu, South Korea, reviewed research into ASM’s role in these conditions. ASM levels increase with stress, bacterial infections and age. Studies on mice suggest high levels of ASM in plasma may disrupt the blood-brain barrier. The team highlight the therapeutic potential of ASM inhibition.

## Introduction

Aging, defined as the progressive decline in physiological function with time, is generally characterized by a reduced ability to cope with stress, failure to maintain homeostasis and increased risk of various diseases, including cancer, and cardiovascular and neurological diseases^1,2^. In particular, alterations in cellular and molecular mechanisms in the central nervous system (CNS) due to aging lead to abnormal aggregation of proteins, neuroinflammation, neuronal death, and cognitive deficits. These aging-related dysfunctions in the CNS lead to neurodegenerative diseases such as mild cognitive impairment, cerebrovascular disease, amyotrophic lateral sclerosis (ALS), Parkinson’s disease (PD) and Alzheimer’s disease (AD)^3–6^. Various hallmarks of aging are associated with the pathogenesis of neurodegenerative disease^2,7^. For example, genomic instability, altered intercellular communication, epigenetic alterations, mitochondrial dysfunction, abnormal protein synthesis, and cell senescence contribute significantly to the aging process and affect the pathology of neurodegenerative diseases^1,2,7^. In addition, previous studies have reported that slight changes in sphingolipid metabolism could be intimately related to aging and age-related neurodegenerative diseases. Thus, sphingolipid abnormalities are increasingly becoming recognized as potential causes of several aging-related neurodegenerative diseases^8–16^. Recently, blood sphingolipids have been considered to be possible biomarkers for these diseases^17–19^.

Sphingolipids are bioactive molecules that include sphingomyelin, ceramide, sphingosine, and sphingosine-1-phosphate (S1P) and are produced through enzymatic pathways. These sphingolipid mediators and their enzymes regulate aspects of cellular and tissue homeostasis, such as the cell cycle, senescence, proliferation, migration, immune response, and inflammation^20–22^. In the CNS, these mediators are highly enriched in the brain, where they are pivotal components of cell membranes and play an essential role in proper brain development and function^23–25^. Defects in sphingolipid metabolism result in disturbances in membrane organization, suppression of cell growth and promotion of apoptosis in different cell types, including neurons^24–27^. Acid sphingomyelinase (ASM), which is encoded by the *Smpd1* gene, is one of the significant sphingolipid-metabolizing enzymes^28^. Two forms of ASM originating from the *Smpd1* gene have been reported: an intracellular lysosomal form and an extracellular secreted form. Based on its localization, secretory ASM is particularly associated with disease^28,29^. The primary role of ASM is to catalyze the conversion of sphingomyelin, a significant component of membranes, into ceramide and phosphocholine^28–30^. In addition, ASM is involved in multiple signaling processes, including cell survival, permeability, proliferation, and differentiation but is also vital in mediating senescence, apoptosis, and autophagy^31–36^.

The importance of ASM has been extensively viewed in several neurodegenerative diseases. Typically, mutations in the *Smpd1* gene induce type A and type B forms of the lysosomal storage disorder Niemann-Pick disease (NPD)^37^. Recently, many studies have shown that the activity or expression of ASM is abnormal in age-related diseases^38–44^. Thus, some reports have demonstrated that the molecular mechanisms correlate with altered ASM levels and pathologies in aging or age-related diseases. In this review, we focus on recent studies that describe the association between ASM and its involvement in aging and age-related neurodegenerative diseases. Furthermore, we discuss the role of ASM as a potential therapeutic target that could have a significant impact on anti-aging and the treatment of neurodegenerative diseases.

## Role of ASM in aging

ASM is expressed in virtually all cell types and is located within the endosomal/lysosomal compartment under normal conditions^30^. ASM can also be preferentially transported to the outer leaflet of the cell membrane and secreted into the extracellular space during cellular stress and disease^29,30^. Previous studies have reported that the expression and activity of ASM change with age^45–47^. Thus, in this section, we review the critical roles of ASM in association with aging-related dysfunctions in physiological processes.

### ASM in aged brain

A recent study revealed that ASM activity was increased in the brain compared to that of other tissues in young, healthy mice, and ASM levels were significantly higher in the brains of old mice rather than in young mice^45^. Such marked elevation in ASM levels in the brains of old mice was associated with microvessels. In particular, increased ASM was mainly derived from endothelial cells (ECs) in the brain. ECs are one of the cell types that compose the blood–brain barrier (BBB), along with pericytes, astrocytes, and other neuronal cells. The BBB has tightly sealed cell-to-cell contacts and restricts entry of most blood-derived molecules and immune cells into the brain^3,48^. In addition, the interaction between ECs and other neuronal cells is critical for the maintenance and regulation of neurological health in the brain^3,48^. Numerous reports have demonstrated dysfunction of ECs in the aged brain, resulting in BBB breakdown^3,48–51^. Previous data also showed that the ASM/ceramide system was involved in the regulation of cell apoptosis and permeability^39,44^. Thus, elevated ASM activity in ECs in old murine brains causes an increase in apoptotic ECs and BBB permeability, while *Smpd1*^*+/–*^ old mice, with genetically reduced ASM, exhibited restoration of such microvessel impairment^45^. BBB hyperpermeability is related to the death of ECs^48–50^, and excessive accumulation of ceramide metabolized by ASM affects cell apoptosis^39,44^. Although ASM-mediated apoptosis of ECs was regulated by p53 apoptotic signaling, apoptotic ECs in the brain did not exhibit vessel leakage in either old control mice or old *Smpd1*^*+/–*^ mice. Moreover, there were no differences in ceramide levels in microvessels between these two types of mice. These observations indicate that increased EC-derived ASM directly affects BBB hyperpermeability in old mice^45^.

Lipid transport and lipid mediators, including ASM, regulate the assembly of caveolae, which are involved in transcytosis across the BBB, at plasma membranes in brain ECs^52,53^. Caveolae are submicroscopic vesicles that are associated with the plasma membrane and consist of caveolin-1 (Cav-1) membrane proteins and cytoplasmic cavin proteins^54–56^. Caveolae are cholesterol-rich and sphingolipid-rich membrane domains and are highly abundant in mechanically stressed cells, such as muscle cells, fibroblasts and ECs^57–59^. Old mice showed a marked increase in caveolae-mediated transcytosis in brain ECs. However, such transcytosis was prevented by genetic inhibition of ASM in the aged brain^45^, highlighting that ASM-regulated caveolae-mediated transcytosis is a pivotal mechanism for regulating BBB permeability in aging. Caveolae transcytosis is regulated by ezrin/radixin/moesin (ERM) proteins, which interact with the actin cytoskeleton at the plasma membrane^58,60–62^. For example, dephosphorylation of ERM proteins by activation of protein phosphatase (PP) contributes to disorganization of the actin cytoskeleton and leads to increases in caveolae internalization^62^. In addition, recent studies have described sphingolipid-mediated ERM activation:^63^ S1P induces ERM phosphorylation and binding to the plasma membrane through phosphatidylinositol biphosphate^64^. In contrast, ceramide produced by ASM activates PP2A to induce ERM dephosphorylation and detachment from the cell membrane^63,65^. Moreover, ASM directly affects ERM dephosphorylation by inducing the activation of PP1^45^. Microvessels derived from old murine brains exhibited significantly increased ERM dephosphorylation, which was improved by normalization of ASM activity^45^. This suggests that EC-derived ASM increases caveolae internalization by cytoskeleton disruption through PP1-mediated ERM dephosphorylation, leading to increased BBB permeability in the aged brain.

Some studies have demonstrated that BBB disruption causes neurodegeneration^48,49,66^. Interestingly, brain EC-specific ASM overexpression induced BBB disruption and further induced a reduction in neuronal cells and severe memory impairment in mice^45^. These findings indicate that brain endothelial-derived ASM accelerates BBB dysfunction and might promote aging-like brain pathology. In addition, this study showed that inhibiting brain endothelial ASM activity improved these pathologies, suggesting that inhibition of brain endothelial ASM may be a highly valuable strategy for anti-aging. Although this result indicated that endothelial ASM mediates BBB dysfunction by increasing caveolae transcytosis in aged murine brains, future studies will need to address whether similar disruption of BBB integrity by endothelial ASM occurs in the aged human brains.

### ASM in aged plasma

Intriguingly, a recent study noted that ASM activity increased significantly in plasma from older versus younger individuals^45^. Similarly, old rodents also showed high levels of ASM activity in the plasma compared to that of younger animals^45,46^. This aging-associated increase in plasma ASM is probably related to ASM secreted from ECs in the brain or peripheral blood vessels. While the specific roles of ASM in the brain have been reported, little is known about the pathogenic mechanisms of abnormal ASM activity in the blood associated with aging. The blood contains various immune cells that originate from bone marrow and circulate throughout blood vessels in the body. Several studies have reported that aged blood and bone marrow showed abnormalities in immune cell distribution^67,68^. The involvement of ASM bioactivity in regulating the functions of innate and adaptive immune cells has recently been explored. After bacterial infection, ASM bioactivity in macrophages induces inflammatory signals and cytokine production^69,70^. Suppression of ASM activity reduces inflammatory cytokine production by macrophages and increases disease resistance^71,72^. ASM is also linked with other immune cell functions, such as T cell differentiation. In particular, ASM is involved in the determination of T helper (Th) cell responses and regulatory T (Treg) cell functions through interactions with cell surface receptors^73,74^. However, most of these roles are mediated by ceramide that is generated by ASM. The increase in ASM activity induces disturbed sphingomyelin degradation and accumulation of ceramide in cell membranes, such that ceramide activates downstream signals associated with Th cell responses or Treg cell function^74^. Sphingosine and S1P, which are downstream sphingolipid mediators of ceramide, regulate cell death and lymphocyte migration, respectively^20,22^. Previous studies showed that aged blood presented a decreased lymphocyte population and defective migration into the thymus for lymphocyte maturation^67,68^. Although there are few reports on the cell-specific levels of sphingosine and S1P by ASM-derived ceramide increases, the accumulation of ceramide affects the levels of sphingosine and S1P in immune cells, and it might be associated with the impairment of lymphocyte function in aged blood.

Many researchers have studied ASM-mediated immune cell function, and it has been noted that by generating ceramide, ASM mediates intracellular signaling. However, whether ASM directly regulates immune cell function is mostly unexplored. Therefore, mechanistic studies to define the pivotal roles of ASM in immune cell regulation are needed, and further research about how altered ASM in the plasma affects aging-related pathologies will provide a greater understanding of the functional role of ASM.

### ASM in aged hearts

ASM secretion is highly related to stressed cells, including ECs and muscle cells. Thus, increased ASM in these cells affects the progression and pathology of numerous diseases associated with vascular dysfunction, such as cardiovascular disease^39^. The prevalence of cardiovascular diseases increases dramatically with age. Notably, disturbances in sphingolipid metabolism in the heart are considered a prerequisite for the development of age-related cardiac pathologies^47,67,75^. Changes in the ASM/ceramide system are also one of the main contributors to age-related cardiac dysfunction. Researchers observed increased ASM activity and ceramide levels in mitochondria isolated from muscle tissue in aged rat hearts^47,67^. Aging-related increases in mitochondrial ceramide levels caused a decrease in cardiolipin content, which leads to mitochondrial dysfunction and contributes to the development of myocardial infarction, stroke and heart failure^76^. ASM suppression by some antidepressant drugs in old rats caused increased cardiac cardiolipin levels^47^, suggesting that the interrelation between the ASM/ceramide system and cardiolipin could be a new therapeutic approach to counteract cardiac dysfunction during aging.

**Fig. 1:**
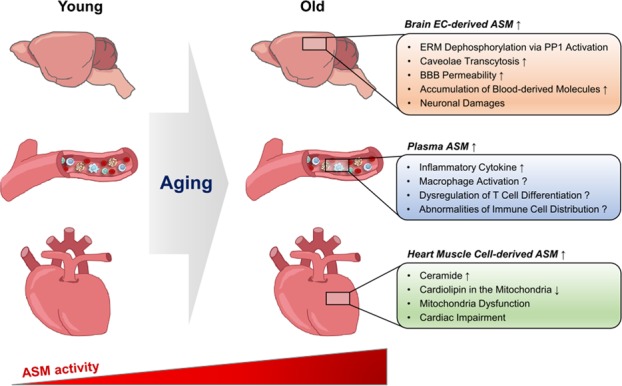
ASM-mediated physiological dysfunction in aging. ASM activity increases with aging. In the aged brain, ASM levels increase in ECs of the BBB. EC-derived ASM induces BBB hyperpermeability by excessive caveolae transcytosis via PP1-mediated ERM dephosphorylation. This results in extravasation of blood-derived molecules into the brain parenchyma, leading to neuronal cell death and memory impairment. Plasma ASM levels also increase with aging, and ASM is likely to promote inflammation by causing immune cell dysfunction, such as macrophage activation and dysregulation of T cell differentiation. In aged hearts, increased ASM in muscle cells induces ceramide production. Ceramide mediates a decrease in mitochondrial cardiolipin, and this reduction contributes to mitochondrial dysfunction and cardiac impairment.

Overall, these findings highlight that ASM increases with age and may be deeply implicated in pathophysiological aging processes (Fig. 1). This hypothesis is also supported by the observation of greater increases in ASM activity in the spleen, liver and muscles of old rodents compared to those in the respective tissues of younger animals^45,67^. Although further studies are needed focusing on the molecular mechanisms of increased ASM in these tissues and plasma under the aging environment, these findings strongly suggests that ASM is important therapeutic target for anti-aging.

## Role of ASM in aging-related neurodegenerative diseases

Aging is the most significant risk factor in neurodegenerative diseases. Some age-related neurodegenerative diseases, including AD, PD and major depression, are also associated with increased ASM levels^39–44^. Below, we highlight recent research into increased ASM and its potential consequences in these age-related neurodegenerative diseases (Table 1).

**Table 1:** ASM-mediated pathologies in age-related neurodegenerative disorders.

Disorder	ASM	Tissue/cell	Functional effects	Refs.
Alzheimer’s disease	Increase	Plasma Neurons Fibroblasts	Reduction of lysosome biogenesis Abnormal autophagic degradation process Aβ accumulation Cognitive impairment	^40,77^
Parkinson’s disease	No change	Plasma Fibroblasts	—	^40^
	Increase	Plasma CNS tissues	Ceramide-mediated Altered lysosomal function Cognitive impairment	^81,82^
Major depression	Increase	Plasma Brain	Ceramide-mediated Neuronal loss Impaired neurogenesis	^47,87–89^
Amyotrophic lateral sclerosis	Increase	Motor neurons	Ceramide-mediated Neuronal apoptosis	^41,44^
Cerebral ischemia	Increase	Astrocytes	Ceramide-mediated Inflammatory cytokine production Neuronal apoptosis	^41,44^
Multiple sclerosis	Increase	Astrocytes	Ceramide-mediated Inflammatory cytokine production Impairment of BBB Increased leukocyte infiltration	^42^

### ASM in Alzheimer’s disease

The specific role and molecular mechanism of ASM have been demonstrated in AD brain pathologies^40,77^. As expected, AD patients had significantly increased plasma ASM levels compared with those of healthy aged individuals. Moreover, 9-month-old amyloid precursor protein/presenilin 1 double mutant (*APP/PS1*) mice, which are used as a murine AD model, showed increased ASM activity in the brain and plasma. Interestingly, increased ASM in the brains of AD mice was related to neurons and may have been due to the stress response that is associated with the progression of AD-like pathologies, such as the accumulation of amyloid β-peptide (Aβ) toxic aggregates in these animals. ASM activity in age-matched *APP/PS1/Smpd1*^*+/–*^ mice was significantly normalized in the plasma, brain, and neurons compared with that of *APP/PS1* mice. Surprisingly, the restoration of ASM levels improved major AD brain pathologies, such as Aβ accumulation, and further dysfunction in learning and memory^40^.

Aβ accumulation in the brain is a significant feature that influences progressive memory loss in AD. Autophagy is an intracellular self-degradation process that mediates intracellular homeostatic turnover of proteins and organelles by regulated degradation and recycling of misfolded or accumulated proteins and damaged organelles^78^. Recent evidence demonstrates that sphingolipids are essential mediators of autophagy; that is, the autophagosome fuses with the lysosome to form the autolysosome^79^. Dysregulated autophagy results in the accumulation of proteins inside the cell, causing cell death, and various autophagy dysfunctions are markedly impaired in AD. Increased autophagosome accumulation has been demonstrated in AD, and studies revealed that such accumulation was due to dysregulated autophagy protein degradation rather than excessive induction of autophagy^40,80^.

Regarding the association between ASM and autophagy, ASM treatment in human neurons enhanced ASM uptake into lysosomes from the extracellular space via mannose-6-phosphate receptor (M6PR). ASM further contributed to autophagosome accumulation due to deficiencies in the autophagic degradation process. The ASM-induced dysfunction in autophagic degradation was mediated by transcription factor EB (TFEB), which is a central regulator of the autophagy-lysosome pathway and lysosome biogenesis^40^. ASM-treated neurons had a significant reduction in TFEB and TFEB target gene expression in the nuclear compartment. In addition, ASM downregulated the lysosomal structural protein LAMP1. Similarly, *APP/PS1* mice showed a marked decrease in TFEB and LAMP1 expression in brain neurons, although this was counteracted by genetic ablation or pharmacological inhibition of ASM^40^. Therefore, increased ASM activity in AD contributes to brain pathology through abnormal autophagic degradation, and restoration of ASM effectively blocks AD progression by ameliorating autophagy, suggesting that ASM inhibition is a potential new therapeutic intervention for AD patients.

### ASM in Parkinson’s disease

The exact mechanism by which ASM is involved in PD pathology remains controversial. One study reported that the activity of ASM was not increased in PD-derived samples compared with that of normal samples^40^. However, some researchers suggested that altered ASM activity was linked to an increased risk of PD^81,82^, although this effect was likely mediated via ceramide because patients with PD with cognitive impairment had increases in several species of ceramides in the plasma compared to that of controls^82^. These results suggest that lipid mediators that are involved in ceramide metabolism, such as ASM, might also be altered in PD patients, particularly in patients with cognitive impairment. Another study also demonstrated the possibility of ceramide involvement in the pathogenesis of PD^83^. Another trial confirmed that ASM expression was markedly increased in patients with PD, which might affect lysosomal dysfunction^81^. Thus, ASM may be directly involved in PD pathology, prompting further investigation focusing on the direct correlation between ASM levels and PD pathology.

### ASM in major depression

There is some evidence that elderly people have a poorer course of major depression that is often associated with deficits in social and cognitive functions^84–86^. Moreover, the role of ASM in depression has been widely investigated for several years. A previous clinical study revealed increased ASM and ceramide levels in patients with major depression^43,87^. Other researchers confirmed depressive-like phenotypes in healthy ASM-overexpressing transgenic (ASMtg) mice of a young age^88,89^. These mice had increased ASM activity and ceramide production in the hippocampus, and enhanced ceramide in this region caused neuronal loss and impaired neurogenesis. In contrast, ASM knockout mice had reduced ceramide levels in the brain and restored depression-related behaviors^88^. In addition, the administration of antidepressant drugs in ASMtg mice improved these abnormal behaviors by decreasing ceramide concentrations^88^. Ceramide generated by ASM also mediates most of these effects, and so whether ASM directly regulates these effects requires further investigation. Interestingly, although ASMtg mice showed depression-related behaviors, these mice did not show cognitive dysfunction, such as memory impairment^89^. This means that other triggers, such as aging factors, are involved in inducing memory dysfunction that is associated with depression due to increased ASM and ceramide. Brain endothelial ASM-overexpressing mice in middle age but not young age showed acceleration of memory dysfunction^45^, supporting this possibility. Therefore, future research is needed to determine whether ASM mediates cognitive dysfunction with depression in age-related depressive disorder.

Since ASM is abundant in the brain and increases with age, ASM-mediated physiological dysfunctions are considered to contribute significantly to the onset of aging-related neurodegenerative disease. As observed in AD, PD, and major depression, other age-related neurodegenerative diseases, such as ALS, cerebral ischemia, and multiple sclerosis, also show significant increases in ASM levels in neuronal tissue (Table 1). Upregulated ASM levels enhance ceramide generation and lead to the production of inflammatory cytokines, destabilization of myelin and neuronal membranes or apoptotic cell death in these diseases^41,42,44^. Further, pharmacological blockade of ASM activity and ceramide accumulation improved these neurodegenerations^42,90,91^, spurring interest in the development of ASM inhibitors.

## ASM as a potential target for therapeutic intervention

**Fig. 2:**
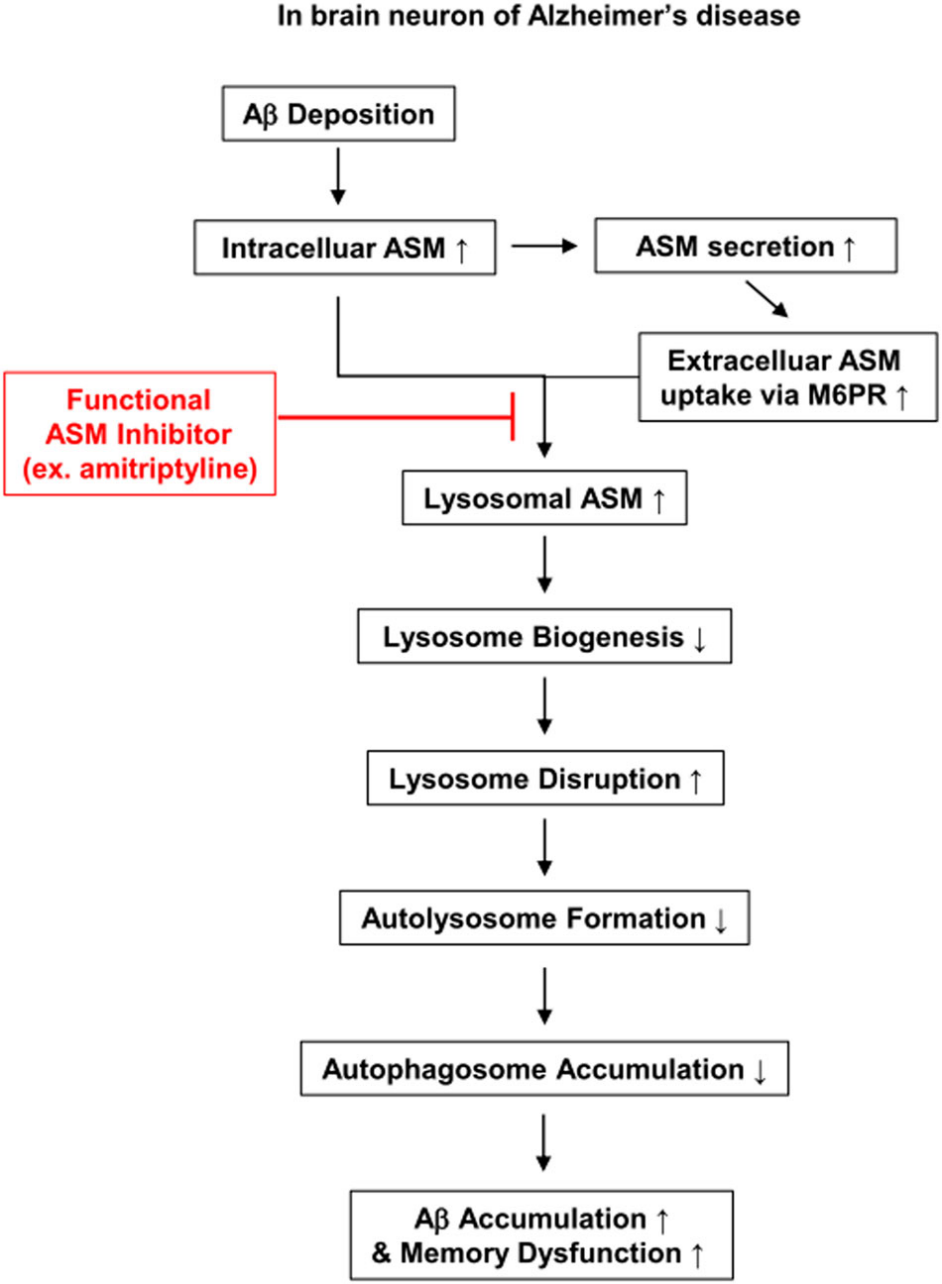
Schematic summary of ASM-mediated pathophysiology and therapeutic effects of ASM inhibition in AD. In AD, ASM is increased in neurons by environmental or cellular stress. Intracellular and secreted ASM can be taken up into the lysosome via M6PR. Excessively increased lysosomal ASM affects lysosomal disruption, and intracellular ASM decreases lysosome biogenesis. This lysosomal disruption by ASM inhibits autophagic protein degradation and further leads to the accumulation of autophagosomes and abnormal proteins, such as Aβ and cytotoxic proteins. Eventually, autophagic flux decreases and induces Aβ deposition and memory impairment in AD. ASM inhibition by functional inhibitors such as amitriptyline blocks AD progression by ameliorating the autophagic process.

As described above, ASM has potential as a drug target for aging and various age-related neurodegenerative diseases. Moreover, the importance of ASM inhibition has already been established in several animal models^32,39,43,88^. In particular, ASM-mediated pathophysiology and the therapeutic effects of ASM inhibition were well established in the AD mouse model (Fig. 2). Despite the significance of ASM, potent and selective inhibitors for this enzyme are rare^92–94^. A few direct ASM inhibitors have been identified, but the systemic availability of these molecules remains unclear (Table 2). Some studies have suggested that several antidepressant drugs and other cationic amphiphilic drugs affect ASM as functional inhibitors^95–99^ (Table 2). These agents inhibit ASM through an indirect, functional mechanism. ASM is mainly anchored to the inner lysosomal membrane via electrostatic forces. These agents are weak bases and accumulate in acidic lysosomal compartments because they become protonated in acidic environments. These positively charged agents alter the electrostatic properties of the inner lysosomal membrane, resulting in detachment of ASM. Upon detachment from the membrane, ASM is cleaved and degraded within the lysosome^97^. In studies aimed at reducing ASM, drugs such as amitriptyline or imipramine ameliorated neurodegeneration in murine models of aging, AD or depression^40,45,88^. However, amitriptyline or other available indirect inhibitors of ASM have some significant disadvantages, such as lack of specificity and the potential for off-target effects. This suggests the need for the rational development of compounds that block ASM by direct interaction. Recently, the organization of the central regions of ASM was described by determining the crystal structure of mammalian ASM in various conformations^100^. This study provides a broad platform for the rational development of ASM inhibitors through a better understanding of the molecular mechanisms of ASM function. Thus, highly potent and selective direct ASM inhibitors are expected to be developed soon and will likely become robust therapeutic agents for anti-aging and the treatment of age-related neurodegenerative diseases.

**Table 2:**
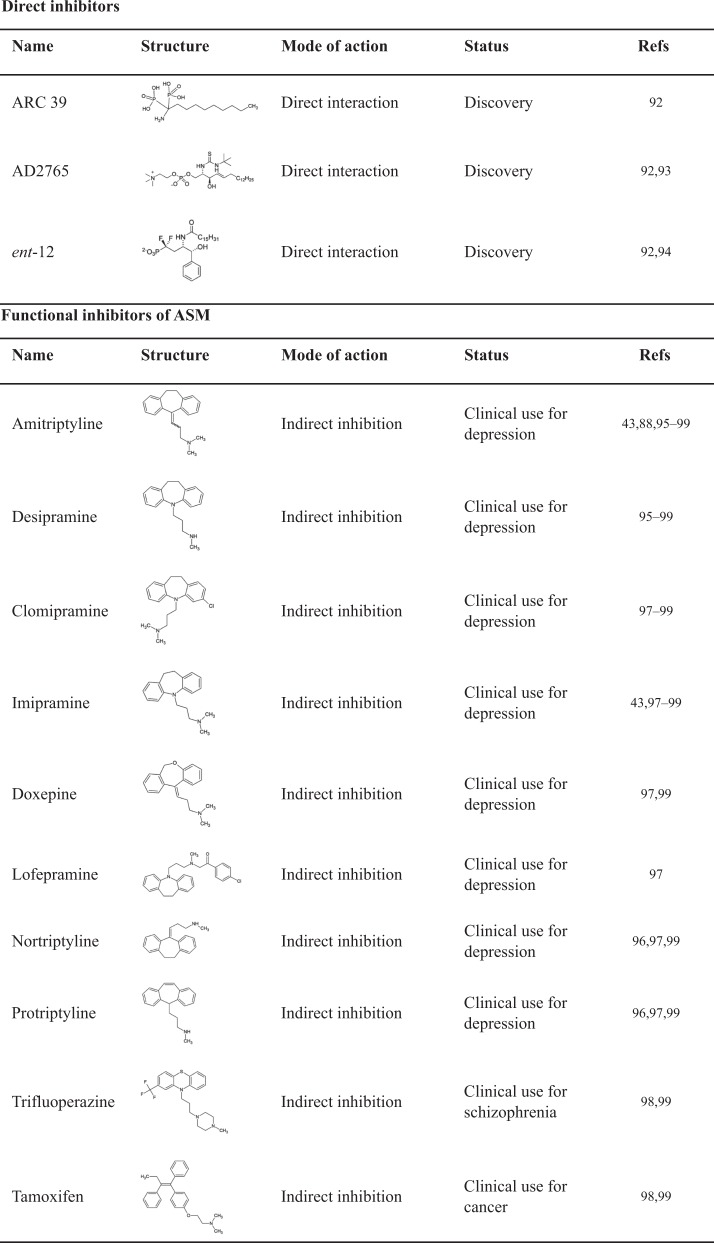
Representative direct inhibitors and functional inhibitors of ASM.

## Future directions

Based on animal experiments, further investigation is needed to confirm the role of ASM in the human setting of aging and age-related neurodegenerative diseases. Moreover, subsequent research focusing on the onset of neurodegenerative diseases from the perspectives of aging and altered ASM levels will likely provide new information that ASM is a critical link between aging and neurodegenerative diseases. Recently, bacterial infections and various stressors have been shown to increase activation and secretion of ASM in the blood, and ASM regulates the functions of blood-derived immune cells^73,74^. Thus, studies determining how ASM alterations in the blood affect various inflammatory disease pathologies will help to clarify the functional role of ASM.
